# Assessment of the Immunogenicity and Safety of an Inactivated Associated Vaccine Against Influenza and Newcastle Disease

**DOI:** 10.3390/vaccines14030248

**Published:** 2026-03-07

**Authors:** Lespek Kutumbetov, Balzhan Myrzakhmetova, Gulzhan Zhapparova, Talshyn Tlenchiyeva, Ayan Tuyakov, Karina Bissenbayeva, Aruzhan Smagulova

**Affiliations:** Research Institute for Biological Safety Problems, Gvardeiskiy 080409, Kazakhstan; lespek.k@gmail.com (L.K.); g.zhapparova@biosafety.kz (G.Z.); t.tlenchiyeva@biosafety.kz (T.T.); asemgul.baygazina@bk.ru (A.T.); k.bissenbayeva@biosafety.kz (K.B.); 18725aruka@gmail.com (A.S.)

**Keywords:** avian influenza, Newcastle disease virus (NDV), inactivated combined vaccine, associated vaccine, aluminum hydroxide gel

## Abstract

Background/Objectives: Combined vaccination against avian influenza (A/H5N3, A/H7N7) and Newcastle disease is of practical interest for reducing handling during immunization and for achieving timely protection in poultry. The aim of this study was to evaluate an inactivated combined (associated) vaccine containing antigenic variants of avian influenza viruses A/H5N3 and A/H7N7 and Newcastle disease virus (NDV). The vaccine is protected by Patent No. 87417. Methods: Viruses with initial reproductive titers of 10^7.5^ EID_50_/mL were inactivated with formaldehyde and formulated as mono-, bi-, or trivalent combinations. Antigens were adsorbed onto aluminum hydroxide gel (1.5%). Immunogenicity was assessed in chicks naïve to avian influenza and Newcastle disease using hemagglutination inhibition (HI) antibody kinetics. Vaccination was performed twice with a 21-day interval. Group administration via drinking water (5 mL/bird) was compared with parenteral administration (1.0 mL/bird). Protective efficacy was evaluated by challenge with virulent viruses at day 30. Sterility and safety/reactogenicity were assessed, and immunobiological performance was additionally evaluated under household farm conditions (337 chickens). Results: Following vaccination, protective immunity was observed starting from day 14. HI titers peaked by day 30 (7.6–7.8 log_2_ for A/H5N3 and A/H7N7; 9.2 log_2_ for NDV) and remained detectable through 180 days (4.3–4.7 log_2_ for avian influenza antigens; 5.1 log_2_ for NDV). Group administration via drinking water produced antibody kinetics comparable to parenteral vaccination, and vaccinated birds were resistant to challenge at day 30. The tested batches met sterility requirements and showed acceptable safety/reactogenicity in laboratory studies. Conclusions: The developed inactivated combined vaccine induced HI antibodies and protective immunity against avian influenza (A/H5N3, A/H7N7) and Newcastle disease. The formulation concept supports flexible antigen combinations and enables group administration via drinking water, which may reduce handling compared with separate vaccinations.

## 1. Introduction

Poultry farming in the Republic of Kazakhstan, as in many other countries, includes industrial enterprises producing eggs, meat, down, and feathers for commercial purposes, as well as small-scale backyard or household farms aimed at meeting the poultry product needs of a single family. These production systems play an important role in meeting the population’s demand for poultry products in the country [1,2,3,4,5,6]. Over recent years, Kazakhstan’s industrial poultry sector has strengthened its capacity to supply the domestic market: domestic production covered about 99% of national demand for table eggs in 2024, while poultry meat self-sufficiency reached about 82% in 2025 [7,8,9]. Further development of the sector requires not only continuous improvement of production technologies, but also effective control of high-impact infectious diseases in poultry populations. Among the most important viral infections are Newcastle disease and avian influenza [10,11,12,13].

Newcastle disease is widely distributed, and its causative agent is maintained and disseminated by various species of wild synanthropic and migratory birds. Epizootics among domestic poultry species are often characterized by almost 100% morbidity, and mortality may reach 90% or higher [14,15,16]. Avian influenza poses a comparable threat to poultry production and is caused by highly pathogenic influenza A viruses comprising multiple antigenic subtypes. Subtypes such as H1N1, H5N1, H7N7, H9N2 and others have been widely reported, and historical data indicate the potential public health risk associated with some subtypes, including H1N1 and H5N1 [17,18,19,20].

To maintain freedom from Newcastle disease and to control this disease, immunoprophylaxis using live and inactivated vaccines (monovalent, associated, and polyvalent) is employed at virtually all industrial poultry enterprises in most countries worldwide. In contrast, systematic vaccination in backyard and individual household farms is, with rare exceptions, not implemented, which contributes to the frequent occurrence of Newcastle disease in such farms and constitutes a significant risk of introduction into industrial poultry production.

For the specific prevention of avian influenza among industrially reared poultry, inactivated mono- and polyvalent vaccines containing antigenic materials of different subtypes have been developed in various countries [21,22,23]. These include monovaccines against the H5N1 subtype produced in Russia [24,25]. In a number of countries, inactivated vaccines based on H5Nx (H5N1/H5N2/H5N8) strains, as well as combined vaccines (e.g., H5/H7), have been described and/or registered, for example in Korea, China, the USA, Germany, France, Italy, Indonesia, and Mexico [23,26,27,28,29,30,31,32,33], and in Kazakhstan [34]. In the Netherlands, the Nobilis Influenza inactivated vaccine line (Intervet/MSD) includes formulations containing antigens of avian influenza A virus subtypes H5N2, H7N7 and H9N2 and may contain one or two strains, enabling both monovalent and bivalent vaccine options [35,36,37]. However, conventional inactivated vaccination programs are characterized by high labor intensity due to the individual method of administration and may therefore increase the overall cost of flock immunization. In addition, the information presented indicates that, in veterinary and poultry practice, prevention of avian influenza and Newcastle disease, including specific prevention and the means for it, has not yet been considered in an integrated manner.

Mass vaccination of poultry by individual injection is labor-intensive and may increase handling-related stress in birds, which complicates large-scale immunization programs. Group administration via drinking water represents a practical alternative for flock-level vaccination. However, while drinking-water delivery is widely used for live vaccines, published data on drinking-water immunization with inactivated poultry vaccines remain limited. Therefore, in this study, we developed and evaluated an inactivated associated vaccine against avian influenza (H5N3, H7N7) and Newcastle disease and compared drinking-water administration with conventional parenteral routes in terms of safety, reactogenicity, immunogenicity, and challenge protection.

The conducted patent and literature search showed that vaccines against Newcastle disease and the most epizootically relevant subtypes of avian influenza virus are currently absent in the veterinary practice of the Republic of Kazakhstan. In view of the identified situation, we set the goal of determining the feasibility of developing a manufacturing process for an inactivated combined vaccine against avian influenza and Newcastle disease. The aim of the studies was to develop and evaluate an inactivated associated vaccine against influenza subtypes A/H5N3, A/H7N7 and Newcastle disease with respect to technological and immunobiological parameters.

## 2. Materials and Methods

### 2.1. Ethical Approval

All animals used in this study were owned and housed by the Research Institute for Biological Safety Problems (RIBSP), Committee of Science, Ministry of Education and Science of the Republic of Kazakhstan. All experimental procedures involving animals were conducted in accordance with applicable institutional and national regulations for animal welfare and with the European Convention for the Protection of Vertebrate Animals Used for Experimental and Other Scientific Purposes (Strasbourg, 1986). The study protocol was reviewed and approved by the Local Committee on Biological Ethics of RIBSP (Protocol No. 1; Permit No. 1408/023; 7 January 2021). All efforts were made to minimize animal suffering and to reduce the number of animals used.

### 2.2. Viruses

In the studies, the “LPB-5/3” strain of subtype H5N3 and the “LPB-7/7” strain of subtype H7N7 of influenza A virus, and the “Almaty-03” strain of Newcastle disease virus (NDV), with infectious activities of 10^4.5^ EID_50_/cm^3^, 10^5.25^ EID_50_/cm^3^, and 10^7.25^ EID_50_/cm^3^, respectively, were used. All viral strains used in this study were provided by the Microorganism Collection Laboratory, Research Institute for Biological Safety Problems (RIBSP), Gvardeyskiy, Kazakhstan.

The avian influenza virus (AIV) strains used in this work correspond to deposited strains held by LLP “KazSRVI”: H5N3 “LPB-5/3” (AV-0001; Kazakhstan Innovation Patent No. 22287) and H7N7 “LPB-7/7” (AV-0003; Kazakhstan Innovation Patent No. 22288). The Newcastle disease virus (NDV) strain “Almaty-03” corresponds to AV-0016 (Kazakhstan Innovation Patent No. 24521). According to the cited patent descriptions, the AIV strains were derived from epizootic avian influenza virus material provided through the FGBI “ARRIAH”, Russian Federation. The NDV “Almaty-03” strain was derived from a field epizootic isolate obtained from a chicken carcass and subsequently cloned by limiting-dilution passages in embryonated eggs. Viral genotype/clade information was not determined in this study.

For propagation of these virus strains, developing chicken embryos (DCE), chicken embryo fibroblasts (CEF) (Laboratory of Cell Biotechnology, Research Institute for Biological Safety Problems (RIBSP), Gvardeyskiy, Kazakhstan), chickens, and chicks were used. To obtain CEF, Eagle’s medium containing 10% bovine blood serum, a 0.25% trypsin solution, Hanks’ working solution, an 8% sodium bicarbonate solution, benzylpenicillin sodium salt, and streptomycin (Thermo Fisher Scientific, Waltham, MA, USA; Gibco, Grand Island, NY, USA) was used.

Virus identification was confirmed by reverse transcription quantitative polymerase chain reaction (RT-qPCR). Viral RNA was extracted from the supernatant of the culture medium using the innuPREP Virus DNA/RNA Kit (IST Innuscreen GmbH, Berlin, Germany). The study was performed using the genesig^®^ Standard Real-Time RT-PCR Detection Kit for avian influenza virus type A (AIV) subtype H5, the genesig^®^ Standard Real-Time RT-PCR Detection Kit for AIV subtype H7, and the genesig^®^ Standard Real-Time RT-PCR Detection Kit for Newcastle disease virus (NDV) (PrimerDesign, Chandler’s Ford, Hampshire, UK) according to the manufacturer’s instructions. Reactions were carried out on a QuantStudio 5 Real-Time PCR System (Thermo Fisher Scientific, Waltham, MA, USA) using specific primers and probes to detect the corresponding genomic targets based on fluorescence signal.

Cycle threshold (Ct) values within the accepted diagnostic range confirmed the presence of the corresponding viral genome. Each run included positive and negative controls to ensure specificity and exclude contamination.

### 2.3. Virus Inactivation

Virus inactivation was performed with formaldehyde. A 10% working formaldehyde solution (GOST 1625-89) was added to the viral biomaterial sample to be inactivated, in various volumes depending on the study objective, and it was held at 37–38 °C for up to 96 h. During warming, every 6–18 h, the studied biomaterial with formaldehyde was mixed for 10–15 s by rocking or shaking. After completion of the inactivation time, the tested samples were cooled at 4–6 °C and used in subsequent studies.

### 2.4. Determination of Completeness of Virus Inactivation

The completeness of inactivation of viral material samples was determined by testing their infectivity and pathogenicity in susceptible biological models. For this purpose, the virus sample treated with formaldehyde for the specified time was administered subcutaneously and intranasally to chicks, inoculated into the allantoic cavity (AP) of 10–11-day-old DCE, and onto a monolayer of CEF cell culture grown in penicillin flasks. Chicks infected with the formaldehyde-treated virus were kept in cages with an appropriate feeding and watering regimen, with daily clinical examination; DCE and CEF were incubated at 37–38 °C, with daily candling of embryos and microscopic examination of the cell-culture monolayer. The presence of infectious virus was determined by morbidity and death of chicks, death, and the appearance of viral hemagglutinin in the allantoic fluid (AF) of RKE, and cytopathic effect (CPE) in CEF. The absence of signs of virus reproduction, manifested by pathogenicity, cytopathogenicity, and embryopathogenicity, indicated completeness of inactivation.

### 2.5. Determination of Vaccine Safety

Safety of the vaccine preparation was tested in chicks and rabbits by intramuscular administration of a 5-fold increased dose of the preparation compared with that recommended for immunization and was assessed based on the clinical condition of these animals and birds over 10 days. For this purpose, 10 chicks not younger than 30 days and rabbits with a live weight of 2.0–2.5 kg were administered the vaccine intramuscularly at 2.5 and 5.0 cm^3^, respectively, and were observed for 10 days under a complete feeding ration and free access to water. Harmfulness of the vaccine was judged by morbidity and death of the observed animals and birds. The absence of disease and death among the animals and birds indicated the safety of the tested preparation.

### 2.6. Adsorption of Viruses onto AHA Particles

Adsorption of viruses onto AHA (GOST 15990-70) was carried out in order to increase the immunogenic activity of the viruses. For this purpose, aluminum hydroxide gel, previously sterilized by autoclaving at 1.0 atm for 60 min, was added to the formaldehyde-inactivated virus at a concentration of 1.0–1.2% by dry residue relative to the total volume of the viral mass. The mixture was thoroughly mixed and left at 8–10 °C for 24 h. After the exposure, the virus adsorbed on the AHA gel was tested in the corresponding required studies.

### 2.7. Determination of Vaccine Reactogenicity

Vaccine reactogenicity was tested in chicks and rabbits by intramuscular administration of the preparation at doses of 0.5 cm^3^ and 1.0 cm^3^, respectively. The indicator was assessed by local and general reactions was assessed based on local and systemic reactions in vaccinated animals and birds detected within 10 days after vaccination.

### 2.8. Determination of Vaccine Immunogenicity

Vaccine immunogenicity was assessed (i) by the presence and levels of specific antihemagglutinin in the sera of vaccinated birds measured by the hemagglutination inhibition (HI) assay and (ii) by the resistance of vaccinated birds to challenge with virulent Newcastle disease virus (NDV) and the corresponding avian influenza virus (AIV) subtypes.

To evaluate immunogenicity, seven groups of birds aged 30–60 days were formed (*n* = 10 per group). Before immunization, blood samples were collected and sera were tested for baseline antiviral antibodies by HI. The first three groups were vaccinated, whereas the remaining four groups served as non-immunized controls. At 21 days post-vaccination, vaccinated birds and the corresponding control groups were challenged as follows: (1) vaccinated group 1 and control group 1 were challenged with NDV at a dose of 10^3^ LD_50_ per bird; (2) vaccinated group 2 and control group 2 were challenged with AIV subtype H5N3 at a dose of 10^3^ LD_50_ per bird; and (3) vaccinated group 3 and control group 3 were challenged with AIV subtype H7N7 at a dose of 10^3^ LD_50_ per bird.

The HI assay was performed using 1% rooster erythrocytes prepared ex tempore. Sera were heat-inactivated at 56 °C for 30 min and adsorbed with rooster erythrocytes to remove nonspecific hemagglutination inhibitors. Twofold serial dilutions of sera were prepared in microtiter plates, after which 4 hemagglutinating units (4 HAU) of the corresponding viral antigen (as determined by prior HA titration) were added. After incubation at room temperature for 30 min, 1% rooster erythrocytes were added, and plates were incubated for an additional 40 min. The HI titer was defined as the highest serum dilution that inhibited hemagglutination (≥2 points on a four-point scoring system) and was expressed as log_2_ values.

The challenge dose (10^3^ LD_50_ per bird) was determined by titration of the corresponding virus in chicks using 10-fold serial dilutions (four birds per dilution), and the 50% endpoint (LD_50_) was calculated using the Reed–Muench method. After the challenge, birds were observed daily for 14 days. Disease onset was defined as the appearance of clinical signs consistent with Newcastle disease or avian influenza (morbidity) and/or death; the course and severity of clinical manifestations were recorded. Birds reaching humane endpoints were euthanized according to institutional protocols.

The vaccine was considered immunogenic if, in each vaccinated group, at least 80% of birds remained clinically healthy, whereas in each corresponding non-immunized control group, at least 80% of birds developed disease.

### 2.9. Statistical Analysis

The analysis of data was conducted utilizing Microsoft Excel 365. Virus titers (log_10_ TCID_50_/cm^3^) were determined employing the Reed–Muench method. The findings are displayed as mean ± standard deviation. Comparisons among groups (such as species, age, or clinical forms) were assessed using Student’s *t*-test or one-way ANOVA, as applicable. A *p*-value < 0.05 was considered statistically significant.

## 3. Results

### 3.1. Technological Parameters for Manufacturing a Vaccine Against Avian Influenza and Newcastle Disease

The technological production cycle for inactivated vaccines usually consists of: (a) obtaining a biological mass of a specific immunogen (virus) with biological activity sufficient for these purposes; (b) inactivation (elimination) of the infectious properties of the pathogenic agent used as a specific immunogen, with maximal preservation of its antigenic properties; and (c) formation of an immunizing complex consisting of the specific antigenic component and its carrier, or a substance that confers on the antigen the required immunogenicity. Since this work involves developing a combined formulation, at the final stage, it is also necessary to determine the quantitative ratios of the specific components relative to one another. As a rule, this technological scheme ends with an assessment of the immunobiological properties of the resulting complex. In accordance with the presented scheme, the studies were initiated by determining effective technological parameters for obtaining an active biomass of avian influenza viruses and Newcastle disease virus, which will serve as the specific component of the preparation under development.

### 3.2. Effective Technological Parameters for Cultivation and Production of an Active Biomass of Avian Influenza Viruses and Newcastle Disease Virus

The technological parameters for cultivation and production of biomass of influenza viruses and Newcastle disease virus using developing chicken embryos (DCE) include the timing of embryo inoculation, the multiplicity of infection of these objects, the temperature and duration of incubation of infected embryos, as well as the timing of collection of the viral mass. Therefore, in the initial experiments, the most effective timing of inoculation (age, days) of DCE was determined, enabling the production of a larger volume of virus-containing extraembryonic fluid. For this purpose, in parallel experiments, each virus was used separately to inoculate, in one case, 10-day-old embryos, in another case 11-day-old embryos, and in a third case 12-day-old embryos. The infected embryos were incubated at 37–38 °C and an air humidity of not less than 60%, and then, at the time of their death, were cooled to 6–8 °C for 3 h to 18–48 h, and the extraembryonic fluid was examined. The resulting allantoic fluid (AF) was evaluated by its volume in cm^3^, freedom from blood cells, hemagglutinin (HA) content, and virus titer. The data from these studies are presented in Table 1.

As can be seen from the data in Table 1, from embryos inoculated at 10–12 days of age, regardless of the type and taxonomic subtype of the causative agent, at the time of their death, an average of 7.2 cm^3^ to 9.1 cm^3^ of extraembryonic fluid containing the virus of one or another designation was collected. No difference in these mean volumes of collected fluid was identified. No significant difference was also detected in the virus titers in the extraembryonic fluid collected from embryos of different age composition used for virus propagation. The hemagglutinin titer of influenza virus subtype A/H5N3 in the collected extraembryonic fluid samples ranged from 7.3 + 0.9 log_2_ to 8.1 + 0.6 log_2_, subtype A/H7N7 from 8.2 + 0.1 log_2_ to 8.7 + 0.2 log_2_, and Newcastle disease virus from 7.3 + 0.2 log_2_ to 9.3 + 0.2 log_2_, with no significant differences in the numerical values. The infectious titers of the causative agents in the samples of this fluid ranged from 10^6.91^ EID_50_ to 10^7.21^ EID_50_ for influenza virus subtype A/H5N3, from 10^7.7^ EID_50_ to 10^8.6^ EID_50_ for subtype A/H7N7, and from 10^8.2^ EID_50_ to 10^9.2^ EID_50_ for Newcastle disease virus. When calculating the total number of viral particles in EID_50_ per total volume of extraembryonic fluid for each virus and for the group of DCE having a single age status, the highest yield of influenza virus subtype A/H5N3 was observed when 11-day-old DCE were used, influenza virus subtype A/H7N7 when 10- and 11-day-old DCE were used, and Newcastle disease virus when 12-day-old embryos were used. Overall, the data obtained indicate that, to obtain an active viral mass of the virulent causative agent of influenza subtypes A/H5N3 and A/H7N7, as well as the velogenic Newcastle disease virus, in relatively large volumetric quantities with the highest infectious antigenic titers, it is necessary to use DCE with an incubation period of 10–11 days.

The selection of the required age of DCE used for inoculation with viruses made it possible to conduct subsequent studies to determine the multiplicity of infection (infecting dose of virus) for each virus, which contributes to more efficient virus propagation. To establish this parameter, 10–11-day-old DCE were divided into several groups, and a virus of a given dose was used to inoculate embryos in each group. Each group consisted of 4 to 10 embryos. The inoculated embryos were incubated under identical temperature conditions, and at the time of their death, the level of accumulation of each virus was determined separately, after opening them under aseptic conditions and collecting the extraembryonic fluid. The virus titer was assessed by hemagglutinin and by the quantitative level of infectivity in DCE. The data from the conducted studies are presented in Table 2.

As can be seen from the data in Table 2, the titers of hemagglutinating activity and embryo infectious dose of influenza and Newcastle disease viruses propagated in DCE at different multiplicities of infection correlated directly proportionally with the quantitative value of the inoculating agent. At inoculating virus doses from 10 to 1000 EID_50_, influenza virus titers ranged from 10^4.3^ to 10^5.8^ EID_50_ for subtype A/H5N3 and from 10^4.8^ to 10^6.7^ EID_50_ for subtype A/H7N7. The velogenic Newcastle disease virus at such inoculation doses accumulated to titers of 10^6.7^–10^7.7^ EID_50_. The highest titers of all tested viruses were observed when a multiplicity of infection of 10^5^–10^7^ EID_50_ per one DCE was used. In this case, the maximum titers of influenza virus subtype A/H5N3 reached 10^6.3^–10^6.7^ EID_50_, subtype A/H7N7 of the same agent 10^7.3^–10^7.8^ EID_50_, and Newcastle disease virus 10^8.7^–10^9.3^ EID_50_.

The dynamics of changes in virus hemagglutinin titers approximately resembled the dynamics of the increase in their infectious titers, correlating directly proportionally with the multiplicity of infection dose. Relatively high hemagglutinin titers of influenza virus, amounting to 7.5–7.8 log_2_ for subtype A/H5N3 and 8.2–8.4 log_2_ for subtype A/H7N7, as well as 9.7–9.8 log_2_ for Newcastle disease virus, were also observed at embryo inoculation doses of 10^5^–10^7^ EID_50_.

The results thus obtained indicate that, in order to obtain virus-containing extraembryonic fluid with high titers of the propagated agents possessing maximal hemagglutinating titers, it is necessary to use a seed-virus multiplicity of infection within 10^5^–10^7^ EID_50_.

In subsequent studies, according to the sequence of manipulations with embryos during virus propagation in them, the timing of embryo collection enabling the production of active virus with the highest titers was established. For this purpose, a batch of embryos consisting of 30 DCE was infected with the virus and incubated at 37–38 °C, monitoring their physiological condition using an ovoscope every 3–18 h. One part of the embryos in the amount of 6–10 was cooled 12–24 h before their death; a second part of the embryos of the same number was cooled during the period of death; and a third part of the embryos remaining in the batch was collected 12–24 h after death. The extraembryonic fluid of each group of embryos was collected differentially, and their infectious and hemagglutinating titers were determined separately by titration in DCE and in the hemagglutination assay, respectively. The most optimal time for virus collection was considered to be the time at which high values of the required titers were observed. The data from the conducted studies are presented in Table 3.

As can be seen from Table 3, the most productive time point for transferring embryos to cooling is the period of embryo death, during which the hemagglutinin titers of influenza virus subtype A/H5N3 reach, on average, 7.8 log_2_, subtype A/H7N7 of this agent 8.2 log_2_, and Newcastle disease virus 9.8 log_2_. The infectious titers of all three viruses at this time are also relatively high. They were 10^6.7^ EID_50_ for avian influenza virus subtype A/H5N3, 10^7.3^ EID_50_ for subtype A/H7N7, and 10^8.7^ EID_50_ for Newcastle disease virus. The hemagglutinating and infectious titers of the viruses 12–24 h before death and 12–24 h after death were significantly lower than those in the extraembryonic fluid collected from embryos removed from incubation during the period of their death.

In the studies described in Table 4 in parallel, the minimum cooling times for embryos with viruses removed from incubation were determined, allowing extraembryonic fluid to be obtained in maximal quantities and of quality with respect to freedom from blood components. For this purpose, part of the DCE cooled after inoculation with the virus was opened, and the extraembryonic fluid was collected after 3 h and 18–24 h, and then evaluated quantitatively and qualitatively in a comparative manner. The obtained data are presented in Table 4.

As can be seen from the data in Table 4, when extraembryonic fluid was collected 3 h after the start of cooling, the volume of the collected biomass ranged within 4.5–5.2 cm^3^/embryo, and it represented a reddish or red turbid fluid, whereas the allantoic fluid from the same DCE kept under cooling for 18–24 h amounted to 7.2–7.8 cm^3^/embryo in volume and represented a colorless transparent fluid. Comparative assessment shows that the amount of extraembryonic fluid collected from DCE cooled for 18 h is 1.3–1.6 times greater than the volume of such fluid collected 3 h after the start of cooling. The obtained data indicate the effectiveness of the cooling holding period for obtaining virus-containing extraembryonic fluid that is standard in quantitative and qualitative terms.

Thus, based on the obtained study results, it should be concluded that obtaining biomass of influenza viruses of subtypes H5N3 and H7N7 and the velogenic causative agent of Newcastle disease with high infectious and hemagglutinating titers by propagation in developing chicken embryos requires compliance with a specific technological protocol, the parameters of which include:

(i) The age of DCE used for propagation of influenza and Newcastle disease viruses;
(ii) The multiplicity of DCE infection with the viruses;
(iii) The incubation temperature of DCE infected with influenza and Newcastle disease viruses;
(iv) The time point for completion of incubation of DCE with replicating influenza and Newcastle disease viruses;
(v) The cooling exposure of DCE with influenza and Newcastle disease viruses.

The study data showed that influenza and Newcastle disease viruses in the extraembryonic fluid of DCE infected with these agents accumulate to relatively high titers when DCE aged 10 to 12 days are used as the propagation substrate, with a multiplicity of infection equal to 10^5^–10^7^ EID_50_/embryo and incubation of infected embryos at 37–38 °C until the time of death. The largest amount of virus-containing extraembryonic fluid not containing cellular blood elements can be obtained after cooling the embryos at 4–8 °C for 24 h after removal from incubation.

### 3.3. Effective Physicochemical Parameters for Inactivation of Influenza Viruses A/H5N3 and A/H7N7 and Newcastle Disease Virus, Enabling Maximal Preservation of the Antigenic Activity of the Causative Agents

To determine the most effective inactivation parameters, the tested viruses were inactivated by exposure to a chemical reagent, formaldehyde, at different concentrations and with different exposure times. To maintain standard conditions and the best inactivating effect, the reaction mixture was maintained at 37–38 °C with periodic mixing (every 6–18 h). Completeness of virus inactivation was determined by residual embryopathogenicity by inoculating formaldehyde-treated samples into the AC of DCE. The specificity of virus development in DCE was determined by embryo lethality and the presence of the agent’s HA in AF. In the absence of signs of the presence of replicating virus, two additional passages of the AC contents into new DCE were performed. The absence of virus already at the third passage was considered complete inactivation in the initial sample. The results of determining the effective parameters for the inactivation of avian influenza viruses and Newcastle disease virus using formaldehyde are presented in Table 5.

As can be seen from the data in Table 5, all three viruses were inactivated under the action of formaldehyde and positive temperature with approximately similar dynamics. Formaldehyde at a concentration of 0.01% inactivated the viruses within up to 48 h, whereas at inactivant concentrations of 0.05–0.1% the agents lost virulence within up to 24 and 12 h. Along with the loss of replicative capacity, the hemagglutinating activity of the viruses also gradually decreased. The rate of decrease in this parameter up to complete inactivation of the agents was 0.8–1.0 log_2_ for subtype A/H5N3, 0.7–1.2 log_2_ for influenza virus subtype A/H7N7, and 1.3–1.8 log_2_ for Newcastle disease virus. Prolongation of exposure in the presence of formaldehyde after complete virus inactivation led to a further decrease in its hemagglutinating titer. Therefore, at the moment of guaranteed complete virus inactivation, the action of formaldehyde was stopped by its neutralization with sodium thiosulfate at a concentration of 0.02% and cooling at 4–6 °C.

Analysis of the inactivation dynamics of the replicative capacity of the viruses shows that all concentrations of formaldehyde used in the tests can effectively inactivate influenza and Newcastle disease viruses. Elevated formaldehyde concentrations for virus inactivation without substantial loss of their antigenicity require a short exposure time, whereas a decrease in formaldehyde concentration requires an increase in exposure time. In all cases, the antigenic activity of the inactivated viruses is preserved at approximately the same level. Therefore, based on the dynamics of the decrease in hemagglutinating activity of the viruses and the rate of their inactivation, for use in further studies on inactivation of the tested viruses in vaccine preparation, a formaldehyde concentration of 0.05% and an exposure time of 48 h at 37–38 °C were selected.

### 3.4. Effective Form and Concentration of the Adsorbing Adjuvant to Ensure Immunogenicity of Inactivated Influenza Viruses A/H5N3 and A/H7N7 and Newcastle Disease Virus

The effective form and concentration of the adsorbing adjuvant were established in studies with each virus separately. Aluminum hydroxide was used as the adsorbing adjuvant in the form of a 10% suspension meeting the requirements of GOST [15990-70], which visually represents a liquid of somewhat thick consistency and white color that forms a sediment at the bottom upon standing. Upon shaking, the white sediment passes into a homogeneous suspension without noticeable effort.

For use in the studies, a 5% gel was additionally prepared from the 10% aluminum hydroxide suspension by grinding its granules and converting the suspension to a gel state using the “RT-2” device (“Tissue Grinder-2”) at a rotational speed of the cutting knives of 3000 rpm for 20 min. Subsequently, in a comparative aspect, the adsorption efficiency of the gel and suspension forms of aluminum hydroxide with respect to avian influenza and Newcastle disease viruses was studied. After establishing the adsorption parameters, they were tested for immunological efficacy in a mixture with the studied agents.

To prepare the gel form from the aluminum hydroxide suspension, the 10% suspension of the preparation was mixed with physiological saline in equal volume ratios and ground using the above device in the mode described above.

When determining adsorption properties, two samples of each studied virus in the active replicative state at previously established infectivity titers in DCE, divided into two equal parts, gel was added to one part and suspension to the other part to a final concentration of 1% by dry residue, and thoroughly mixed. The resulting mixture was maintained at 4–6 °C for 24 h, centrifuged at 3000× *g* for 30 min, and the supernatant was tested by titration in DCE for the quantitative virus indicator. The amount of adsorbed virus was determined by the difference between the initial and supernatant titer. When determining the titer of the initial virus sample, physiological saline was added to the separated titrated aliquot in a volume proportionally equal to the volume of aluminum hydroxide introduced for adsorption. The results of studies to determine the adsorption capacity of different forms of aluminum hydroxide are presented in Table 6.

As can be seen from the data in Table 6, aluminum hydroxide in the suspension state at an amount of 1.5% of the total mass of the virus-containing fluid adsorbed from 82.21% to 94.37% of the virus, whereas the preparation in the gel form at the same concentration bound from 99.0% to 99.7% of the infectious units of the agents. These data show that aluminum hydroxide in the gel form binds 5–17% more avian influenza and Newcastle disease viruses than its suspension form. No noticeable difference in the amount of adsorbed virus depending on their type was noted.

In studies to verify the immunological efficacy of the suspension and gel forms of aluminum hydroxide, each virus was adsorbed separately; the virus had been previously inactivated with formaldehyde under the regimen selected the virus had been previously inactivated with formaldehyde under the selected regimen (0.05% for 48 h at 37–38 °C), and was administered separately intramuscularly to poultry intact with respect to influenza and Newcastle disease, after previously selecting the sorbent concentration.

Selection of an effective immunological concentration of the sorbent was carried out in an experiment with Newcastle disease virus, which was adsorbed separately with the gel form of aluminum hydroxide taken at concentrations of 1.0%, 1.5%, and 2.0%, and these samples were used to immunize four chicks each by intramuscular inoculation at a dose of 0.5 cm^3^, after previously collecting blood serum samples. After 3 weeks, blood serum was collected from the chicks and examined retrospectively for an increase in antihemagglutinins. The effective sorbent concentration was judged by the level of formed antibodies. The data from these studies are presented in Table 7.

The data in Table 7 indicate that the titers of antibodies to Newcastle disease virus detected in the blood serum of vaccinated chicks increase in proportion to the concentration of the sorbent in the viral mass. The antibody titers observed at an adjuvant concentration of 1.5 and 2.0% were 0.75–1.0 log_2_ higher than at a concentration of 1.0%; therefore, these concentrations are more preferable for inducing a strong immune response in the organism. The adjuvant at concentrations of 1.5 and 2.0% stimulates antibody production at approximately the same titer; therefore, and in view of the relatively increased viscosity of the suspension obtained when using an AHG concentration of 2%, a sorbent concentration of 1.5% was used for vaccine manufacture.

When assessing the immunological role of the AHG sorbent as an adjuvant, it was added to the virus-containing fluid in an amount of 1.5% by dry residue. Virus adsorption with it was carried out for 24 h at 4–6 °C. The immunological efficacy of the administered virus with the sorbent was evaluated using retrospective data from examination of poultry blood serum collected before administration and several days after injection. The results of these studies are presented in Table 8.

As can be seen from the data in Table 8, antibodies were detected in the blood serum of vaccinated chicks to all three viruses inactivated and adsorbed with the suspension and gel forms of aluminum hydroxide, indicating acquisition of antigenic activity by static viral particles in association with the particulate carrier. The antibody titers to the viruses in a mixture with suspension aluminum hydroxide ranged within 4.75–7.25 log_2_, and in a mixture with the gel form, 5.25–8.50 log_2_. Calculation of the mean antihemagglutinin titers for each group shows that the mean antibody titer in the group immunized with viruses in a mixture with the gel form of aluminum hydroxide (6.83 log_2_) was 1.0 log_2_ higher than that in the group vaccinated in a mixture with aluminum hydroxide suspension (5.83 log_2_). The information obtained as a result of these studies gives preference to the use of the gel form of aluminum hydroxide in preparation of the vaccine product.

Thus, based on the study results obtained in investigating the dynamics of inactivation and concentration by adsorption with aluminum hydroxide, it was established that reliable inactivation with preservation of the hemagglutinating activity of avian influenza viruses (A/H5N3, A/H7N7) and Newcastle disease virus occurs when they are treated with the indicated inactivant at concentrations of 0.05–0.1% at 37–38 °C for 48 h, and virions of these viruses in the greatest amount, reaching 99% and higher, are adsorbed on particles of aluminum hydroxide taken in the gel form at a final concentration of 1.0% by dry residue relative to the virus-containing extraembryonic fluid. The inactivated virions of avian influenza viruses and Newcastle disease virus adsorbed on aluminum hydroxide particles acquire dynamic antigenic activity, which stimulates the formation of a strong immunity in vaccinated poultry. The optimal concentration of aluminum hydroxide used as an adjuvant sorbent of influenza and Newcastle disease viruses to stimulate humoral immunity against diseases caused by these agents is an amount of 1.5–2.0% by dry residue relative to the total volume of the viral suspension.

The antibody titers formed in response to stimulation with aluminum hydroxide gel loaded with influenza and Newcastle disease virus virions increase by one order (1 log_2_) more than the antihemagglutinin titers formed in response to stimulation with the suspension form of this adjuvant with the indicated viruses.

### 3.5. Immunologically Effective Concentrations and Ratios of Inactivated Viral Particles of the Causative Agent of Influenza A/H5N3, A/H7N7 and Newcastle Disease

The objective of the studies in this section was to determine the concentrations of influenza and Newcastle disease viruses that exert a sufficient immunogenic effect in the associated state and the quantitative ratios of these immunogens to each other when mixed for the purpose of vaccine preparation. For this purpose, in two batches of experiments, the studied viruses were used with significantly different initial infectious and hemagglutinating titers. In the first batch of experiments, influenza and Newcastle disease viruses were taken at titers of 10^4.0^–10^5.5^ EID_50_/cm^3^ with hemagglutinating titers of 3–4.5 log_2_, and in the second batch, the infectious titers of the viruses were 100–1000, and the hemagglutinating titers were 3–4-fold higher. The viruses were inactivated according to the regimen identified in Section 2.2 of this work and adsorbed with the gel form of the adjuvant-AHG, with a final concentration of 1.5%. To test the dose-dependent immunological effect, four chicks aged 35 days were immunized with each virus type, and their blood serum samples were examined after 21 days for the presence and level of specific antihemagglutinins, after which a control challenge with virulent viruses at a dose of not less than 10^4^ ELD_50_/head was performed. Immunological efficacy of the viruses used for vaccination was evaluated by the titers of the formed antihemagglutinins and resistance to the virulent virus. The results of these studies are presented in Table 9.

As can be seen from the data in Table 9, in all groups of poultry vaccinated with inactivated viruses with different concentrations before inactivation, specific antihemagglutinins were detected on day 21, and their titers were proportional to the concentrations of the administered viral antigen. In poultry vaccinated with viruses at concentrations of 10^4.00^–10^5.50^ EID_50_ with hemagglutinating titers of 3.0–4.5 log_2_, antihemagglutinin titers on day 21 reached only 2.5–3.0 log_2_; however, upon control challenge, they were resistant to influenza virus subtypes A/H5N3 and H7N7. Despite the presence of antihemagglutinins at a titer of 3.0 log_2_, chicks vaccinated with inactivated Newcastle disease virus at a concentration of 10^5.50^ EID_50_ became ill and died upon control challenge with the homologous virulent virus.

In the analysis of blood serum from poultry vaccinated with inactivated viruses at initial concentrations of 10^6.50^–10^8.25^ EID_50_ with hemagglutinating titers of 6.5–8.2 log_2_, antihemagglutinin titers on day 21 were recorded at the level of 5.5–7.5 log_2_, and upon control challenge with homologous virulent viruses, they were completely resistant. No survivors among the control poultry groups after infection with virulent viruses were recorded.

The data of the conducted studies on establishing immunologically effective virus concentrations show that immunological reactivity develops in the organism of vaccinated poultry, capable of producing specific antibodies and resistance to the homologous virulent agent, the level of which, apart from the adjuvant, depends on the concentrations of the viruses administered for vaccination.

A continuation of these studies comprised experiments whose task included determining the immunological efficacy of inactivated viruses in a mixture with an adjuvant in association with different concentrations. In these studies, virus samples were used that were applied in the tests described in Table 10. In one variant, from samples of inactivated influenza viruses’ subtypes A/H5N3 and A/H7N7 and Newcastle disease virus with initial low titers of 10^4.00^, 10^4.25^ and 10^5.50^ EID_50_, respectively, by addition in equal volume ratios, a first mixture was prepared, which was subsequently used as an associated immunizing preparation. In another variant, from samples of inactivated influenza viruses’ subtypes A/H5N3 and A/H7N7 and Newcastle disease virus with initial increased titers of 10^6.50^, 10^7.25^ and 10^8.25^ EID_50_, respectively, by addition also in equal volume ratios, a second mixture was prepared, which was subsequently tested as an associated immunizing preparation. Each virus, before mixing, was adsorbed with aluminum hydroxide gel (AHG) taken at a concentration of 1.5% by dry residue.

The associated complexes of inactivated influenza virus subtypes A/H5N3 and A/H7N7 and Newcastle disease virus prepared in this way in a mixture with the sorbent-adjuvant were tested in parallel studies for immunological efficacy. For this purpose, three groups of 60-day-old poultry, 16 birds in each, were used. The first group of poultry was vaccinated intramuscularly at a dose of 1.0 cm^3^ with the associated immunizing preparation prepared from samples of influenza viruses subtypes A/H5N3 and A/H7N7 and Newcastle disease virus with initial low titers; the second group of poultry was vaccinated in the same manner and at the same dose with the associated immunizing preparation prepared from samples of the same viruses but with increased initial titers. The third group of poultry was kept as a control without inoculation of viral preparations. Before use, a blood serum sample was collected from each bird in all groups. Each group of poultry was kept in separate rooms under compliance with identical feeding, watering, and housing conditions. At 21 days after vaccination, blood was collected from the entire poultry stock of the three groups to obtain serum, and four chicks from each group were challenged with virulent influenza virus subtype A/H5N3 at a dose of 10^4.0^ ELD_50_, another four chicks from each group with subtype A/H7N7 of the same agent at the same dose, and a third group of four chicks with velogenic Newcastle disease virus at a dose of 10^5.0^ ELD_50_. The remaining four chicks in each group were separated into another isolated room and were subsequently used in other studies aimed at investigating the dynamics of the post-vaccination immune response of poultry.

Based on the results of challenges with virulent viruses and retrospective blood serum examinations, the immunological efficacy of the inactivated tested viruses in association with each other was evaluated. The data from the conducted tests are presented in Table 10.

As can be seen from the data in Table 10, on day 21, specific antihemagglutinins were detected in all vaccinated chicks at titers of 2.3–2.8 log_2_ in the first group and 4.6–6.4 log_2_ in the second group. At the same time, antibodies specific to all three viruses were detected in the blood serum of each bird, indicating that the immune response of the immunocompetent system of the poultry organism in parallel reacts to an equal extent to all three viruses used for vaccination. The recorded titers against the viruses under associative and separate application are uniform and similar, except for the numerical values, which under associative application are relatively lower, explained by the fact that under associative use, 0.15 cm^3^ of each virus was used, whereas under separate use, this dose was 0.5 cm^3^.

Upon infection with virulent viruses, results similar to those in Table 9 were also obtained. All vaccinated birds infected with virulent influenza virus subtypes A/H5N3 and A/H7N7 were protected against these agents, whereas among poultry vaccinated with the associated immunizing preparation from inactivated viruses with initial low titers, Newcastle disease was observed upon infection with the virulent virus. Of four chicks infected with this agent, three became ill and died. However, in these chicks, unlike the control intact ones, the disease manifested slowly, progressed chronically with signs of lesions of the nervous system. In the control group of poultry, upon infection with virulent viruses, both influenza and Newcastle disease manifested within 2–4 days, progressed acutely, and in all cases ended with fatal outcomes. Poultry vaccinated with the associated immunizing preparation produced from viruses with increased titers were completely resistant against all virulent viruses used for control infection.

Thus, the results of the conducted studies indicate that the immunological competence of inactivated avian influenza and Newcastle disease viruses loaded onto aluminum hydroxide particles is proportionally dependent on the concentration titer of the viruses per unit volume of the virus-containing suspension used for adsorption by the adjuvant. Inactivated influenza and Newcastle disease viruses with hemagglutinating titers below 4.5 log_2_, loaded onto aluminum hydroxide particles, stimulate the formation of antihemagglutinins in the organism of vaccinated poultry, but do not exert sufficient immunological impact contributing to development of a strong immunity, whereas these same viruses with hemagglutinin titers of 6.5 log_2_ and higher after inactivation and adsorption with an adjuvant sorbent exert an immunostimulating effect in the organism of vaccinated poultry, as a result of which a strong specific immunity is formed. Inactivated influenza and Newcastle disease viruses adsorbed on aluminum hydroxide in the associative state manifest their immunological competence as well as when used in a monovalent form. Signs of interferogenic interaction between antigens of influenza and Newcastle disease viruses are not manifested.

Based on the obtained data for the group of poultry vaccinated with an association of inactivated influenza viruses subtypes A/H5N3 and A/H7N7 and Newcastle disease virus with increased initial titers in a mixture with aluminum hydroxide gel, the method of preparing this preparation was taken as the basis of the technology for manufacturing an associated inactivated vaccine preparation against influenza subtypes A/H5N3 and A/H7N7 and Newcastle disease.

To prepare experimental vaccine samples, two batches of 10–11-day-old DCE, 60 each, were sequentially used. After appropriate ovoscopy and shell sterilization by flaming with alcohol flame, the embryos were infected separately with avian influenza viruses’ subtypes A/H5N3 and A/H7N7 and Newcastle disease virus. In each of the two batches, 20 DCE were infected with each virus at doses of 10^5.0^–10^7.0^ EID_50_ and incubated at 37–38 °C until the time of death. The infected embryos were observed daily every 3–15 h by ovoscopy. Embryos that died within the first 24 h were discarded; the remaining ones were collected for cooling before death. Dead embryos were cooled at 4–8 °C for 18–24 h, then opened under aseptic conditions; AF was collected with a pipette, and its hemagglutinating and infectious activities were determined. If titers of at least 10^6.5^ EID_50_/cm^3^ for avian influenza virus subtypes A/H5N3 and A/H7N7 and at least 10^8.0^ EID_50_/cm^3^ for the causative agent of Newcastle disease were present, they were used for vaccine preparation. The viruses were inactivated separately with formaldehyde at a concentration of 0.05% by holding for 48 h at 37–38 °C. The inactivated virus was adsorbed with 5% aluminum hydroxide gel (AHG) by adding the adjuvant to the virus suspension in an amount of 1.5% by dry residue and holding at 4–8 °C for 24–48 h. The resulting mixture was then dispensed as 10 cm^3^ into penicillin vials and used in further studies as an experimental vaccine. Data on the preparation of microbatches of the vaccine intended for standardization are presented in Table 11.

As can be seen from Table 11, according to the developed technology, two batches of an inactivated, adsorbed, associated vaccine were prepared, with a volume of 530–540 cm^3^ in each batch, corresponding to 1060–1080 vaccination doses. The obtained vaccine batches were subjected to further studies to test for immunobiological parameters. The list of these parameters included sterility with respect to microbiological and mycological contaminants, harmlessness, reactogenicity, and immunogenicity in poultry.

### 3.6. Sterility of the Inactivated Associated Vaccine Against Avian Influenza and Newcastle Disease

Sterility was assessed according to GOST 28085. The results for two experimental microbatches of the inactivated associated vaccine (A/H5N3, A/H7N7, and NDV) are shown in Table 12.

Table 12 shows that no bacterial or fungal growth was detected in either experimental vaccine batch. Therefore, both batches met sterility requirements.

### 3.7. Harmlessness of the Inactivated Associated Vaccine Against Avian Influenza and Newcastle Disease

Harmlessness of the vaccine was tested in 30-day-old chicks and outbred white mice by intramuscular administration of the product. Ten chicks were administered the vaccine of each batch at a dose of 2.5 cm^3^, which is five times higher than the immunizing dose, and mice were administered 0.3 cm^3^. Vaccinated poultry and mice were observed daily for 10 days, and systemic and local clinical signs were recorded. The observation data for poultry and mice in these studies are presented in Table 13.

As shown in Table 13, intramuscular administration of higher vaccine doses induced local and systemic reactions in chicks, including transient lethargy, reduced appetite and activity, and soreness at the injection site; these effects remained noticeable for 1–3 days. Such changes were noted in 3–5 chicks out of the 10 used. Subsequently, these pathologies disappeared spontaneously without therapeutic intervention. No local or general pathologies were noted in vaccinated mice. Ultimately, all poultry and white mice used to verify vaccine harmlessness remained healthy and alive. The obtained results indicate that the tested vaccine batches, at doses increased by not less than five times compared with the immunizing dose, are harmless for chicks and white mice.

### 3.8. Reactogenicity and Immunogenicity (Determination of the Immunizing Dose, Time to Onset of Immunity, and the Magnitude and Duration of the Immune Response) of the Inactivated Combined Vaccine Against Avian Influenza and Newcastle Disease

Vaccine reactogenicity was determined by local and general reactions of poultry used in the immunogenicity tests of this product, vaccinated intramuscularly and subcutaneously at a dose of 0.5 cm^3^.

Vaccine immunogenicity was tested in 60-day-old chicks. In the first studies, the effective method of immunization and the dynamics of formation of immunocompetent factors-specific antihemagglutinins were determined. For this purpose, eight chicks were used, four of which were vaccinated subcutaneously and the remaining four intramuscularly with the vaccine of one microbatch. From the chicks, before vaccination and at 7, 14, and 21 days, blood serum samples were collected, which were examined for antihemagglutinins against influenza viruses’ subtypes A/H5N3 and A/H7N7 and Newcastle disease virus. Based on the rate of antibody development and antibody titers, we evaluated the effectiveness of the immunization method and determined the time to onset of immunity. The results obtained in the course of the studies are presented in Table 14.

The study results presented in Table 14 show that specific antibodies against the viruses on the basis of which the vaccine was created appear earlier in the blood serum of vaccinated chicks after subcutaneous immunization. Their presence in blood serum is detected on day 7 after subcutaneous vaccination. In chicks vaccinated intramuscularly, such antibodies were detected on day 14 after vaccination. On day 21 after immunization, no noticeable differences are observed in the antihemagglutinin titers detected in the blood serum of poultry vaccinated by the two different methods. The obtained data indicate that the tested immunization methods are equally effective in inducing immunity against avian influenza and Newcastle disease using the tested vaccine.

In the subsequent studies, the number of minimal immunizing doses in one vaccination dose of the manufactured vaccine was determined in order to establish a guarantee of the immunogenicity of the product. These studies were performed using the first microbatch of the product. For this purpose, sequential dilutions of 1:3, 1:6, and 1:9 were prepared from the vaccine using physiological sodium chloride solution, and four chicks were vaccinated intramuscularly with each dilution at a dose of 0.5 cm^3^. After 21 days, blood serum samples were collected from the poultry for testing for the presence of antihemagglutinins and were tested for resistance to infection with virulent influenza virus subtypes A/H5N3 and A/H7N7 and Newcastle disease virus. The study results are presented in Table 15.

As can be seen from the data in Table 15, an immunological effect was noted for all tested vaccine dilutions, which was evidenced by the presence of antihemagglutinins to the tested viruses, except for the last dilution, in which antibodies to Newcastle disease virus were absent.

However, the presence of antibodies up to certain limits of their titers did not guarantee protection of poultry against infection with virulent viruses. Poultry vaccinated with a 1:3 vaccine dilution were fully protected against all three types of virulent viruses, whereas poultry vaccinated with higher vaccine dilutions were not fully resistant to virulent viruses.

The obtained data indicate that each vaccination dose of the vaccine recommended for vaccination contains not less than three minimal immunizing doses against each influenza virus subtype and Newcastle disease virus. Such a number of minimal immunizing doses serves as a guarantee of a reserve of the immunogenic activity of the vaccine.

**Figure 1:**
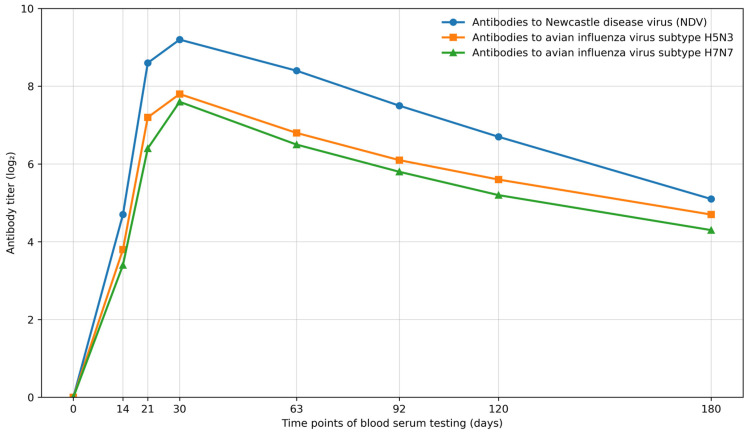
Dynamics of antihemagglutinin titers to influenza virus subtypes A/H5N3 and A/H7N7 and Newcastle disease virus in the blood serum of poultry vaccinated twice with the associated vaccine.

In studies to establish the duration of immunity induced by the tested developed vaccine, a group of 60-day-old chicks (*n* = 6) was vaccinated twice with an interval of 21 days with the second experimental microbatch of the product at a dose of 0.5 cm^3^ intramuscularly and kept for 180 days, examining blood serum samples every 30 days for the presence and level of specific antihemagglutinins to avian influenza viruses subtypes A/H5N3 and A/H7N7 and Newcastle disease virus. The duration of immunity was judged by the presence and level of detected antibodies. The data from these studies are presented in Figure 1.

As can be seen from the data in Figure 1, in the organism of vaccinated poultry, specific antihemagglutinins were formed to all viruses included in the vaccine, the titers of which increased up to 30 days after the first vaccination and by this time reached 7.6 log_2_ to subtype A/H5N3, 7.8 log_2_ to subtype A/H7N7 of avian influenza virus, and 9.2 log_2_ to Newcastle disease virus. In subsequent periods, antibody titers decreased uniformly, and by the end of the study at 180 days after the first vaccination, they amounted to 4.3 log_2_ to subtype A/H5N3, 4.7 log_2_ to subtype A/H7N7 of avian influenza virus, and 5.1 log_2_ to Newcastle disease virus. The detected antibody titers indicate that vaccinated poultry acquired a strong immunity against virulent avian influenza viruses’ subtypes A/H5N3 and A/H7N7 and Newcastle disease, which persisted for 180 days (the observation period).

In all studies on testing the immunogenicity of the associated vaccine, daily clinical observation of the general condition of vaccinated poultry and the injection site was carried out to assess its reactogenicity. As a result, in no case were any pathologies noted in poultry at the vaccine administration site or in their overall clinical condition. These data indicate clinical areactogenicity of the tested associated vaccine with respect to chickens.

### 3.9. Immunogenicity of the Inactivated Associated Vaccine Against Influenza and Newcastle Disease When Administered by Drinking

Inactivated vaccines are predominantly used by individual methods via syringe injections. Individual vaccination methods are labor-intensive and take a long time, especially in poultry farms where large numbers of immunized animals are concentrated. In infectious diseases such as Newcastle disease and highly pathogenic avian influenza, the speed of immunity development is crucial for the timely containment of epizootic outbreaks and the successful eradication of the disease. One possible solution is mass vaccination, in which immunization of the susceptible population is less labor-intensive and can be carried out within a short time. One of such methods is the administration of vaccines to poultry by mixing with drinking water. However, with alimentary immunization methods for most diseases, the active immunity produced is not always strong and forms at more distant times after vaccination. In the literature sources available to us, information on immunization of poultry with inactivated vaccines against influenza and Newcastle disease by drinking administration of the product was not registered, except for products prepared from live attenuated viruses. The aim of the studies was to determine the possibility of inducing immunity against Newcastle disease and highly pathogenic avian influenza using an inactivated vaccine when administered by drinking.

In these studies, we used an inactivated combined vaccine against influenza A/H5N3 and A/H7N7 and Newcastle disease, prepared using the technology described previously. The vaccine was tested in 60-day-old chicks that were intact with respect to influenza and Newcastle disease and had not previously been immunized against these diseases. To determine the possibility of immunizing poultry through drinking and to evaluate its results, three groups of nine birds each were formed. The first group of poultry was given the vaccine in a mixture with drinking water at a dose of 5 cm^3^/head, the second group was administered it subcutaneously at a dose of 1.0 cm^3^/head, and the third group of chicks was used as a control. The timing of formation and the dynamics of specific immunity were determined by antihemagglutinins specific to each virus, and the intensity of immunity by the resistance of vaccinated poultry to control challenge with virulent viruses 30 days after vaccination.

Observation results of vaccinated poultry showed that no pathological changes in their clinical condition were noted throughout the entire experimental period. The data of studies of blood serum of poultry used in the studies for antihemagglutinins specific to influenza viruses A/H5N3 and A/H7N7 and Newcastle disease are presented in Table 16.

As can be seen from the data in Table 16, before vaccination antiviral antibodies were absent in all chicks of the three groups, and on days 7–14 after vaccination, regardless of the method of administration of the product, antihemagglutinins to influenza A virus subtypes H5N3 and H7N7 and the causative agent of Newcastle disease were detected in the blood serum of vaccinated poultry. Analysis of the timing and dynamics of antibody formation shows that in chicks vaccinated by the enteral method, on day 7 after vaccination, antihemagglutinins were noted in six out of nine, whereas in the group vaccinated subcutaneously, at this time, such antibodies were detected only in three chicks out of the same number. On day 14, specific antibodies were noted in all chicks of both vaccinated groups, but antibody titers in the blood serum of poultry vaccinated by the enteral method were 0.3 log_2_ higher than the antibody titers in chicks vaccinated by the subcutaneous method. Probably, the appearance of antibodies at relatively high titers was influenced by the increased dose of the product administered enterally. In blood serum samples collected at subsequent times after vaccination, antihemagglutinins to influenza and Newcastle disease viruses were detected at approximately the same titers.

Upon control challenge with virulent viruses, vaccinated poultry on day 30 after immunization proved resistant to the disease. These data show that immunity in poultry developed as a result of vaccine administration to the organism by enteral and subcutaneous methods is strong and does not differ noticeably between them.

Thus, the results of the conducted studies indicate that the inactivated associated vaccine against influenza and Newcastle disease, when administered by the enteral method, has the capacity to induce immunity against the corresponding diseases equivalent to the immunity produced by the subcutaneous immunization method.

## 4. Discussion

The causative agent of influenza in animals, poultry, and humans is highly diverse and exhibits unpredictable, variable biological properties. This variability is inherent to the viral genetic material and becomes apparent under the influence of certain factors that make survival in the natural ecological niche non-permissive (unfavorable or impossible) [38,39,40,41,42,43,44,45,46]. Therefore, the production and practical use of live influenza vaccines in preventive programs face a number of barriers. First, obtaining live modified vaccine strains requires considerable time and thorough scientific and practical substantiation of their safety and immunogenic efficacy. Second, the circulation of a given subtype variant of the pathogen in each epizootic event is unpredictable; consequently, a live vaccine developed over a long period and with substantial effort may prove ineffective in a particular situation [47,48,49,50,51]. Accordingly, the most effective approach for organizing influenza prevention and control measures is epizootiological monitoring of the components of the epizootic process, genetic and antigenic analysis of circulating pathogen populations, and the pre-emptive use of inactivated vaccines produced from viruses that are homologous or antigenically close to the circulating field variant [49,52,53]. This logic is consistent with current international trends in avian influenza control and is reflected in the vaccine platforms used and the models for vaccine implementation across regions.

Thus, in global avian influenza control practice, inactivated vaccines (typically emulsion-based) are the most widely used, particularly in countries where vaccination is a systemic element of risk management strategies; however, a key limitation remains in the need for regular updates of vaccine antigenic composition in accordance with dominant circulating virus variants (with several publications highlighting China’s experience in updating vaccine strains/antigens and adapting them to currently relevant clades, including clade 2.3.4.4b) [54,55]. In the European Union, the approach is more strongly based on principles of managed vaccination and surveillance, including the concept of differentiating infected from vaccinated animals (DIVA); therefore, recombinant vectored vaccines (e.g., HVT-based vectors expressing H5), such as Vectormune HVT-AIV and Vaxxitek HVT+IBD+H5 (the latter authorized “under exceptional circumstances”), play an important role [30,56,57]. At the same time, injectable H5 products based on other platforms are also being considered in the EU along the regulatory pathway (e.g., EMA publications on the status/CVMP opinions for Vaxxinact H5) [57]. Practical implementation of vaccination in Europe is expanding: in France, duck vaccination was initiated in October 2023, and a published assessment links it to a substantial reduction in the expected number of outbreaks in the 2023–2024 season; however, implementation experience also underscores the need for evidence-based surveillance and consideration of trade and regulatory implications [58,59]. Similarly, a pilot vaccination program for laying hens has been implemented in the Netherlands with restrictions on product circulation, demonstrating a stepwise implementation approach and the dependence of scale-up on monitoring effectiveness and risk management [60,61]. In the United States, a key indicator of a regulatory shift was the USDA issuance of a conditional license for an inactivated H5N2 vaccine for chickens (Zoetis), reflecting the possibility of limited use upon confirmation of safety and a reasonable expectation of efficacy, although decisions on mass vaccination remain part of broader veterinary and policy strategy [27,62]. In contrast to avian influenza, vaccination against Newcastle disease has long been a standard measure and includes live attenuated, inactivated, and recombinant vectored vaccines (e.g., based on HVT or poxvirus vectors), as reflected in WOAH guidance documents [63]. In the EU, this is further supported by the long-standing authorization of recombinant products against Newcastle disease (e.g., Vectormune ND and Inno-vax-ND-IBD), making ND prevention more established compared with the dynamic and more regulatorily sensitive field of H5/H7 avian influenza vaccination [64,65].

In this context, our data showed that avian influenza and Newcastle disease viruses with initial reproductive titers of 10^7.5^ EID_50_/cm^3^, after inactivation and adsorption to 1.5% aluminum hydroxide gel, are capable of inducing strong immunity against the respective diseases starting from day 14 after vaccination when administered either by individual parenteral inoculation or by group enteral methods [66,67,68]. These findings enabled the production, first, of monovalent vaccines prepared separately from each virus type and, subsequently, of an associated vaccine containing all three viruses. Testing of the immunobiological properties of these vaccines showed that, regardless of valency, the preparations induced strong immunity against homologous virulent viruses. The level of humoral immunity detected in the blood serum of vaccinated poultry was approximately the same when monovaccines were used separately and when the associated vaccine was used. Antihemagglutinin titers formed after vaccination with monovaccines against influenza and Newcastle disease ranged from 5.5 to 7.5 log_2_, whereas after vaccination with the associated preparation, they ranged from 4.6 log_2_ to 6.4 log_2_.

In one combined dose of the associated vaccine contained at least three immunizing doses against each disease caused by three virus variants. When using the associated vaccine, no signs of interference in immunogenicity or antigenicity between the three inactivated viruses were observed.

It is widely known that inactivated vaccines are generally administered individually by parenteral inoculation. Although drinking-water vaccination is widely used for mass immunization in poultry, published data on drinking-water delivery of inactivated vaccines remain limited. Therefore, we specifically evaluated the immunogenicity and protective efficacy of our inactivated associated avian influenza–Newcastle disease vaccine when administered via drinking water.

Drinking-water vaccination is a well-established approach for mass immunization in poultry because it reduces labor demands and handling-related stress [69,70]. However, reproducible outcomes require a standardized administration protocol (water quality, correct vaccine preparation, and proper maintenance of the drinking system) to ensure the most uniform possible vaccine intake across birds [71,72]. In contrast, inactivated poultry vaccines are generally administered by injection, and published data supporting drinking-water delivery of inactivated vaccines remain limited [69,73]. In this context, our findings are noteworthy: drinking-water administration of the inactivated associated vaccine induced a systemic humoral response and challenge protection comparable to parenteral immunization. The tendency toward earlier antibody detection after enteral administration may reflect sufficient early antigen exposure, although an effect of the higher volume/dose used for drinking-water delivery cannot be excluded. A plausible explanation is repeated and/or prolonged antigen contact with the oropharyngeal and upper gastrointestinal mucosa during drinking; however, mechanistic interpretation is limited because we primarily assessed systemic immunity by hemagglutination inhibition (HI) and did not evaluate mucosal immune markers (e.g., local IgA) [74,75]. Another limitation is the relatively high oral volume (5 mL per bird), which may affect cost-effectiveness and scalability. Future studies should therefore determine the minimum effective dose/volume, optimize administration protocols to improve uniform intake, and assess vaccine stability in drinking water over practically relevant time intervals.

Consistent with these considerations, our experimental results indicated that antihemagglutinins and resistance to virulent challenge developed after drinking-water administration with kinetics and overall levels comparable to those observed after individual parenteral immunization.

## 5. Conclusions

Testing of additional vaccine quality parameters (sterility, safety, and reactogenicity) showed that all batches produced using the developed technology were free from viable bacterial and fungal contaminants, were harmless in laboratory animals and chicks, and were clinically weakly reactogenic, indicating conformity with the required standards [76,77,78]. The immunobiological properties of the inactivated associated vaccine against avian influenza and Newcastle disease observed in laboratory studies were further supported under household poultry farm conditions involving 337 chickens. No vaccine-associated pathologies were detected in vaccinated birds, and antihemagglutinins to avian influenza and Newcastle disease viruses were detected in sera at titers of 5.0–9.0 log_2_.

Influenza viruses A/H5N3 and A/H7N7 and Newcastle disease virus with initial infectious activity of at least 10^7.0^, 10^7.5^, and 10^8.0^ EID_50_/cm^3^, respectively, when inactivated with 0.05% formaldehyde at 37–38 °C for 48 h and adsorbed onto 1.5% aluminum hydroxide gel (AHG) for 24 h at 4–8 °C, demonstrated pronounced immunogenic properties and may be used as monovalent preparations or combined as an associated vaccine.

The associated inactivated adsorbed vaccine against A/H5N3, A/H7N7, and Newcastle disease induced measurable humoral immunity and protection in chicks older than two months when administered intramuscularly/subcutaneously and, under the experimental conditions tested, when administered via drinking water. Given the relatively high volume used for drinking-water delivery (5 mL per bird) and the absence of mucosal immune measurements in the present study, further work should optimize the minimum effective oral dose/volume and standardize drinking-water administration protocols to improve practical feasibility and uniform intake in flock settings.

## Figures and Tables

**Table 1:** Yield of virus-containing extraembryonic fluid and the virus titer therein of influenza and Newcastle disease viruses when embryos of different ages were used for inoculation.

Object of Inoculation	Age of the Object, Days	Inoculating Virus	Number of Objects, pcs.	Volume of Viral Suspension, Embryo/cm^3^	Virus Titer		Total Amount of Virus, EID_50_
					HA, log_2_	EID_50_	
DCE	10	AI-H5	43	8.5 ± 0.7	8.1 ± 0.6	10^6.91^	69,088,000
		AI-H7	37	7.4 ± 0.4	8.4 ± 0.1	10^7.7^	370,888,000
		ND	32	7.8 ± 0.2	7.3 ± 0.2	10^8.2^	126,800,000
	11	AI-H5	127	8.7 ± 0.5	7.8 ± 0.4	10^7.15^	122,931,000
		AI-H7	148	9.1 ± 0.03	8.7 ± 0.2	10^8.6^	3,622,710,000
		ND	93	8.4 ± 0.12	8.5 ± 0.1	10^8.7^	4,210,080,000
	12	AI-H5	32	7.2 ± 1.1	7.3 ± 0.9	10^7.21^	116,784,000
		AI-H7	27	8.3 ± 0.21	8.2 ± 0.1	10^8.1^	1,044,970,000
		ND	24	8.7 ± 0.07	9.3 ± 0.2	10^9.2^	13,789,500,000

Note: AI: avian influenza; ND: Newcastle disease.

**Table 2:** Infectivity titers and hemagglutinating activity titers of influenza and Newcastle disease viruses propagated in DCE at different multiplicities of infection *n* = 3; *p* < 0.001.

Inoculating Virus: Type, Subtype	Multiplicity of Infection of DCE—Dose, EID_50_									
	10		10^2^		10^3^		10^5^		10^7^	
	HA	EID_50_	HA	EID_50_	HA	EID_50_	HA	EID_50_	HA	EID_50_
AI A/H5N3	3.2	10^4.3^	4.7	10^5.0^	4.3	10^5.8^	7.5	10^6.3^	7.8	10^6.7^
AI A/H7N7	3.7	10^4.8^	5.5	10^5.8^	6.2	10^6.7^	8.4	10^7.8^	8.2	10^7.3^
NDV	4.5	10^6.7^	5.2	10^6.3^	7.8	10^7.7^	9.7	10^9.3^	9.8	10^8.7^

**Table 3:** Infectivity titers and hemagglutinating activity titers of influenza and Newcastle disease viruses in DCE at different collection and cooling times after inoculation.

Inoculating Virus: Type, Subtype, Species	Number of Inoculated DCE, pcs.	Cooling Time Points for Inoculated DCE, Period								
		12–24 h Before Death			During Death			12–24 h After Death		
		Number of DCE, pcs.	Titer Unit		Number of DCE, pcs.	Titer Unit		Number of DCE, pcs.	Titer Unit	
			HAU, log_2_	EID_50_		HAU, log_2_	EID_50_		HAU, log_2_	EID_50_
AI A/H5N3	28	8	4.8	10^4.5^	12	7.8	10^6.7^	8	5.2	10^5.5^
AI A/H7N7	34	10	5.7	10^5.0^	16	8.2	10^7.3^	8	6.2	10^6.7^
NDV (velogenic)	24	7	6.8	10^6.3^	11	9.8	10^8.7^	6	5.4	10^7.3^

**Table 4:** Quantitative and qualitative characteristics of virus-containing extraembryonic fluid of DCE collected at different times after transferring to cooling.

Virus Harvested from Cooled DCE: Type, Subtype, Species	Number of Cooled DCE, pcs.	Time to Opening After Transfer to Cooling, h					
		After 3 h			After 18–24 h		
		Number of DCE, pcs.	Characteristics		Number of DCE, pcs.	Characteristics	
			Volume, cm^3^	Color, Clarity		Volume, cm^3^	Color, Clarity
AI A/H5N3	28	7	4.7 ± 0.5 (33)	Turbid, reddish	21	7.8 ± 1.4 (150)	Colorless, transparent
AI A/H7N7	34	7	4.5 ± 0.8 (32)	Turbid, reddish	26	7.5 ± 1.2 (195)	Colorless, transparent.
NDV (velogenic)	24	5	5.2 ± 1.2 (26)	Turbid, reddish	19	7.2 ± 0.7 (137)	Colorless, transparent

Note—The total volume of DCE extraembryonic fluid is given in parentheses.

**Table 5:** Physicochemical parameters for formaldehyde inactivation of avian influenza viruses and Newcastle disease virus.

Viruses			Formaldehyde Concentration, %	Exposure at 37–38 °C, h				
Name	Volume, cm^3^	Titer, log_2_ HAU		12	24	48	72	96
Avian influenza A/H5N3	200	7.8	0.01	+/7.5	+/7.2	−/6.8	−/5.4	−/5.2
			0.05	+/7.3	−/7.0	−/6.5	−/6.5	−/6.0
			0.1	−/6.8	−/6.5	−/6.0	−/6.0	−/6.0
Avian influenza A/H7N7	250	8.2	0.01	+/8.0	+/7.6	−/7.4	−/7.0	−/6.5
			0.05	+/7.5	−/7.0	−/6.5	−/6.0	−/6.0
			0.1	−/7.5	−/6.5	−/6.0	−/6.0	−/6.0
Newcastle disease	250	9.8	0.01	+/9.5	+/8.5	−/8.5	−/8.0	−/7.5
			0.05	+/9.0	−/8.0	−/7.5	−/7.0	−/7.0
			0.1	−/8.5	−/8.0	−/8.0	−/7.0	−/6.5

Note: “+” indicates that a replicating virus was detected; “−” indicates that no replicating virus was detected.

**Table 6:** Adsorption capacity of aluminum hydroxide with respect to avian influenza viruses and Newcastle disease virus.

Adsorbent		Virulent Virus		Titration Steps		Adsorption Capacity of the Adsorbent	
Form and Concentration, %	Volume, cm^3^	Type, Subtype, Species	Volume, cm^3^	Virus Titer Before Adsorption, log_10_ EID_50_/mL	Virus Titer After Adsorption, log_10_ EID_50_/mL	EID_50_	%
5% aluminum hydroxide suspension	15	AIV H5N3	35	6.50	5.75	3.06 × 10^6^	82.21
	15	AIV H7N7	35	6.75	5.75	5.06 × 10^6^	90.00
	15	NDV	35	8.50	7.25	2.98 × 10^8^	94.37
5% aluminum hydroxide gel	15	AIV H5N3	35	6.50	4.50	3.13 × 10^6^	99.0
	15	AIV H7N7	35	6.75	4.75	5.57 × 10^6^	99.0
	15	NDV	35	8.50	6.00	3.15 × 10^8^	99.7

**Table 7:** Antihemagglutinin titers to inactivated Newcastle disease virus administered in a mixture with aluminum hydroxide gel (AHG) at different concentrations.

Inactivated Virus		Adsorbent Adjuvant		Number of Vaccinated Chicks, Head	Time Points of Examination	
Name	HA Titer, log_2_	Name, Form	Concentration in the Viral Mass, %		Before Vaccination	After 21 Days
ND	7.5	5% aluminum hydroxide suspension	1.0	4	0	6.50 ± 0.21
			1.5	4	0	7.25 ± 0.34
			2.0	3	0	7.50 ± 0.17

**Table 8:** Antihemagglutinin titers in the blood serum of poultry vaccinated with influenza and Newcastle disease viruses, inactivated and adsorbed on aluminum hydroxide.

Adsorbent		Virus Inactivated		Number of Vaccinated Chicks, Head	Time Points for Testing Chick Blood Serum	
Form and Concentration, %	Volume, cm^3^	Type, Subtype, Species	Volume, cm^3^		Before Vaccination	After 21 Days
5% aluminum hydroxide suspension	30	AIV H5N3	70	4	0	4.75 ± 0.12
	30	AIV H7N7	70	4	0	5.50 ± 0.07
	30	NDV	70	4	0	7.25 ± 0.34
5% aluminum hydroxide gel	30	AIV H5N3	70	4	0	5.25 ± 0.23
	30	AIV H7N7	70	4	0	6.75 ± 0.17
	30	NDV	70	4	0	8.50 ± 0.14

Note: Antihemagglutinin titers are given as log_2_ of twofold serial dilutions of blood serum.

**Table 9:** Immunological efficacy of inactivated avian influenza and Newcastle disease viruses depending on their concentrations in a mixture with aluminum hydroxide gel (AHG).

Group	Virus (Inactivated, Adsorbed)			Number of Vaccinated Birds, Head	AHA Titer in Blood Serum, log_2_		Challenge Infection			
	Type	Activity (Initial)					Virus		Results	
		Infectious, EID_50_	HAU, log_2_		Before Vaccination	After 21 Days	Type	Dose, ELD_50_	Became Ill, Head	Survived, Head
1	AI H5	10^4.00^	3.0	4	0	2.5	AI H5	10^4^	0	4
2	AI H7	10^4.25^	4.2	4	0	3.0	AI H7	10^4^	0	4
3	ND	10^5.50^	4.5	4	0	3.0	ND	10^5^	4	0
4	AI H5	10^6.50^	6.5	4	0	5.5	AI H5	10^4^	0	4
5	AI H7	10^7.25^	7.4	4	0	6.4	AI H7	10^4^	0	4
6	ND	10^8.25^	8.2	4	0	7.5	ND	10^5^	0	4
7	Control	-	-	3	0	0	AI H5	10^4^	3	0
8	Control	-	-	3	0	0	AI H7	10^4^	3	0
9	Control	-	-	3	0	0	ND	10^5^	3	0

**Table 10:** Immunological efficacy of inactivated avian influenza and Newcastle disease viruses in association, depending on their concentrations in a mixture with aluminum hydroxide gel (AHG).

Group	Virus (Inactivated, Adsorbed)			Number of Vaccinated Birds, Head	AHA Titer in Blood Serum, log_2_		Challenge Infection			
	Type	Activity (Initial)					Virus		Results	
		Infectious, EID_50_	HAU, log_2_		Before Vaccination	After 21 Days	Type	Dose, ELD_50_	Became Ill, Head	Survived, Head
1	AI H5	10^4.00^	3.0	16	0	2.3	AI H5	10^4^	0/4	4/4
	AI H7	10^4.25^	4.2		0	2.7	AI H7	10^4^	0/4	4/4
	ND	10^5.50^	4.5		0	2.8	ND	10^5^	3/4	1/4
2	AI H5	10^6.50^	6.5	16	0	4.6	AI H5	10^4^	0/4	4/4
	AI H7	10^7.25^	7.4		0	5.2	AI H7	10^4^	0/4	4/4
	ND	10^8.25^	8.2		0	6.4	ND	10^5^	0/4	4/4
3	Control	-	-	16	0	0	AI H5	10^4^	4/4	0/4
4	Control	-	-		0	0	AI H7	10^4^	4/4	0/4
5	Control	-	-		0	0	ND	10^5^	4/4	0/4

**Table 11:** Results of preparation of experimental vaccine batches.

Vaccine Batch, №	Viruses: Type, Subtype, Species	Quantity, Virus Titer			Inactivation Procedure	Adsorption Procedure	Vaccine Volume, cm^3^
		Volume, cm^3^	HAU, log_2_	Infectious, EID_50_	Formaldehyde	AHG	
1	AI A/H5N3	150	7.0	10^7.0^	0.05%, 48 h.	1.5%, 24–48 h.	(410 + 120) = 530
	AI A/H7N7	140	7.4	10^7.5^	0.05%, 48 h.	1.5%, 24–48 h.	
	ND	120	8.2	10^8.5^	0.05%, 48 h.	1.5%, 24–48 h.	
2	AI A/H5N3	130	7.2	10^7.0^	0.05%, 48 h.	1.5%, 24–48 h.	(420 + 120) = 540
	AI A/H7N7	150	7.8	10^7.5^	0.05%, 48 h.	1.5%, 24–48 h.	
	ND	140	8.7	10^8.5^	0.05%, 48 h.	1.5%, 24–48 h.	

**Table 12:** Results of sterility testing of the vaccine against influenza subtypes A/H5N3 and A/H7N7 and Newcastle disease.

Vaccine Batch, №	Passage, №	Microbiological and Mycological Culture Media			
		MPA	MPB	MPLB	SDA
1–2	1–3	0/4	0/4	0/4	0/4
	1–3	0/4	0/4	0/4	0/4

Abbreviations—MPA, meat–peptone agar; MPB, meat–peptone broth; MPLB, meat–peptone liver broth; SDA, Sabouraud dextrose agar. Note—the denominator indicates the number of inoculated tubes; the numerator indicates the number of tubes positive for contamination.

**Table 13:** Results of harmlessness testing of the vaccine against influenza subtypes A/H5N3 and A/H7N7 and Newcastle disease in chicks and white mice.

Vaccine		Test Subjects		Test Results		
Batch, №	Dose, cm^3^	Type	Number, Head	Presence of a Reaction		Survived, Head
				Local, Head	Systemic, Head	
1	2.5	Chicks	10	3 *	3	10
	0.3	White mice	5	-	-	5
2	2.5	Chicks	10	2	4	10
	0.3	White mice	5	-	-	5

Note: “-” indicates absence of a reaction; “*” indicates the number of animals and birds exhibiting a reaction.

**Table 14:** Dynamics of antibody formation to influenza and Newcastle disease viruses in the blood serum of chicks vaccinated with the vaccine by different methods.

Vaccine Batch, №	Vaccine Administration Route	No. of Vaccinated Chicks, Head	Antibodies to Viruses	Study Time Points, Days			
				0	7	14	21
1	s/c	4	AI virus A/H5N3	0	1.5	4.5	7.0
			AI virus A/H7N7	0	1.0	4.0	7.5
			NDV	0	0.5	4.0	8.5
	i/m	4	AI virus A/H5N3	0	0	3.0	6.5
			AI virus A/H7N7	0	0	2.5	7.0
			NDV	0	0	3.5	7.5
2	s/c	3	AI virus A/H5N3	0	1.5	4.5	7.0
			AI virus A/H7N7	0	1.0	4.0	7.5
			NDV	0	0.5	4.0	8.5
	i/m	3	AI virus A/H5N3	0	0	3.0	6.5
			AI virus A/H7N7	0	0	2.5	7.0
			NDV	0	0	3.5	7.5

**Table 15:** Resistance of poultry vaccinated with the diluted associated inactivated vaccine to virulent influenza viruses and Newcastle disease virus.

Vaccine Batch, №	Vaccine Dilution	Number of Vaccinated Birds, Head	Antibodies			Challenge with Virulent Viruses	
			To Viruses:	At Time Points, Days			
				0	21	Causative Agent	Mortality (Dead/Total Challenged)
Vaccine against AI and ND, batch № 1	1:3	9	AI virus A/H5N3	0	4.5	AI virus A/H5N3	0/3
			AI virus A/H7N7	0	5.4	AI virus A/H7N7	0/3
			NDV	0	5.2	NDV	0/3
Vaccine against AI and ND, batch № 1	1:6	9	AI virus A/H5N3	0	2.2	AI virus A/H5N3	1/3
			AI virus A/H7N7	0	3.4	AI virus A/H7N7	0/3
			NDV	0	2.3	NDV	3/3
	1:9	6	AI virus A/H5N3	0	1.4	AI virus A/H5N3	1/2
			AI virus A/H7N7	0	0.5	AI virus A/H7N7	2/2
			NDV	0	0	NDV	2/2
Control	placebo	6	AI virus A/H5N3	0	0	AI virus A/H5N3	2/2
			AI virus A/H7N7	0	0	AI virus A/H7N7	2/2
			NDV	0	0	NDV	2/2

Note: the denominator indicates the number of challenged birds, and the numerator indicates the number of birds that became ill and died.

**Table 16:** Antihemagglutinin titers to influenza viruses A/H5N3 and A/H7N7 and Newcastle disease virus in blood serum samples of poultry vaccinated with the inactivated associated vaccine against influenza and Newcastle disease by enteral and subcutaneous methods.

Poultry		Antigenic Components of the Vaccine, Virus Type	Time Points for Collection of Poultry Blood Serum, Days					Virulent Viruses		
Group, №	Number, Head		0	7	14	21	30	H5N3	H7N7	ND
1-vaccinated enterally	9	AI virus A/H5N3	0	1.7	2.4	4.3	3.7	0/3	0/3	0/3
		AI virus A/H7N7	0	2.1	3.3	4.5	4.1			
		NDV	0	1.9	2.3	5.1	4.3			
2-vaccinated subcutaneously	9	AI virus A/H5N3	0	0.3	2.1	4.1	4.2	0/3	0/3	0/3
		AI virus A/H7N7	0	0.2	2.7	4.7	4.5			
		NDV	0	0.4	1.9	4.8	4.4			
3-unvaccinated (control)	9	-	0	0	0	0	0	3/3	3/3	3/3
		-	0	0	0	0	0			
		-	0	0	0	0	0			

Note: AI virus: avian influenza virus; NDV: Newcastle disease virus; antibody titers are given in log_2_; the denominator indicates the number of challenged chicks, and the numerator indicates the number that became ill.

## Data Availability

The original contributions presented in this study are included in the article. Further inquiries can be directed to the corresponding author.
